# Population genomics of free‐ranging Great Plains white‐tailed and mule deer reflects a long history of interspecific hybridization

**DOI:** 10.1111/eva.13330

**Published:** 2021-12-14

**Authors:** Fraser J. Combe, Levi Jaster, Andrew Ricketts, David Haukos, Andrew G. Hope

**Affiliations:** ^1^ Division of Biology Kansas State University Manhattan Kansas USA; ^2^ Kansas Department of Wildlife and Parks Topeka Kansas USA; ^3^ Department of Horticulture and Natural Sciences, Wildlife and Outdoor Enterprise Management Kansas State University Manhattan Kansas USA; ^4^ Division of Biology U.S. Geological Survey Kansas Cooperative Fish and Wildlife Research Unit Kansas State University Manhattan Kansas USA

**Keywords:** conservation genomics, double‐digest restriction‐site associated DNA sequencing, genetic diversity, migration, single nucleotide polymorphisms, wildlife management

## Abstract

Hybridization is a natural process at species‐range boundaries that may variably promote the speciation process or break down species barriers but minimally will influence management outcomes of distinct populations. White‐tailed deer (*Odocoileus virginianus*) and mule deer (*Odocoileus hemionus*) have broad and overlapping distributions in North America and a recognized capacity for interspecific hybridization. In response to contemporary environmental change to any of one or multiple still‐unknown factors, mule deer range is contracting westward accompanied by a westward expansion of white‐tailed deer, leading to increasing interactions, opportunities for gene flow, and associated conservation implications. To quantify genetic diversity, phylogenomic structure, and dynamics of hybridization in sympatric populations of white‐tailed and mule deer, we used mitochondrial cytochrome b data coupled with SNP loci discovered with double‐digest restriction site‐associated DNA sequencing. We recovered 25,018 SNPs across 92 deer samples from both species, collected from two regions of western Kansas. Eight individuals with unambiguous external morphology representing both species were of hybrid origin (8.7%), and represented the product of multi‐generational backcrossing. Mitochondrial data showed both ancient and recent directional discordance with morphological species assignments, reflecting a legacy of mule deer males mating with white‐tailed deer females. Mule deer had lower genetic diversity than white‐tailed deer, and both mitochondrial and nuclear data suggest contemporary mule deer effective population decline. Landscape genetic analyses show relative isolation between the two study regions for white‐tailed deer, but greater connectivity among mule deer, with predominant movement from north to south. Collectively, our results suggest a long history of gene flow between these species in the Great Plains and hint at evolutionary processes that purge incompatible functional genomic elements as a result of hybridization. Surviving hybrids evidently may be reproductive, but with unknown consequences for the future integrity of these species, population trajectories, or relative susceptibility to emerging pathogens.

## 1 INTRODUCTION

A central issue in modern wildlife management is understanding how species will respond to ongoing and future environmental change (Quilodrán et al., 2020; Todesco et al., 2016). The contemporary demography and distribution of species are increasingly affected by anthropogenic stressors including habitat fragmentation, industrial/urban development, long‐term exploitation, and accelerating global climate change (Allendorf & Hard, 2009; Bellard et al., 2012; Fahrig, 2003). Recent population dynamics can be interpreted through genetic legacies that may reveal biogeographic connectivity, demographic trends, and associated evolutionary trajectories. Evolutionary change includes fluctuating genetic diversity, neutral genetic drift, adaptive selection, and gene flow within species through dispersal and between species through periodic hybridization (Cutter & Payseur, 2013; Edwards et al., 2016). A molecular approach to monitoring and model‐based population assessments of biodiversity may therefore significantly enhance the information gained from more traditional field‐based estimation and experimentation, toward more effective and holistic applied wildlife management. For instance, analysis of genetic connectivity can reflect the role of landscape features and land‐use practices for constraining or promoting species‐range shifts, immigration/emigration among populations, and interspecific interactions (Fenderson et al., 2020). Continued declines in sequencing costs and rapid advances in bioinformatics have made comparative population genomics widely accessible across non‐model wildlife. Genetic demographic data are now integrated for addressing multiple priorities, including maintenance of diversity (reflecting fitness), long‐term monitoring of population trends, understanding disease transmission, assessing adaptation to local environments, and understanding the role of hybridization in species differentiation (Allendorf et al., 2010; Andrews et al., 2016; Forester et al., 2020; Storfer et al., 2020).

**FIGURE 1:**
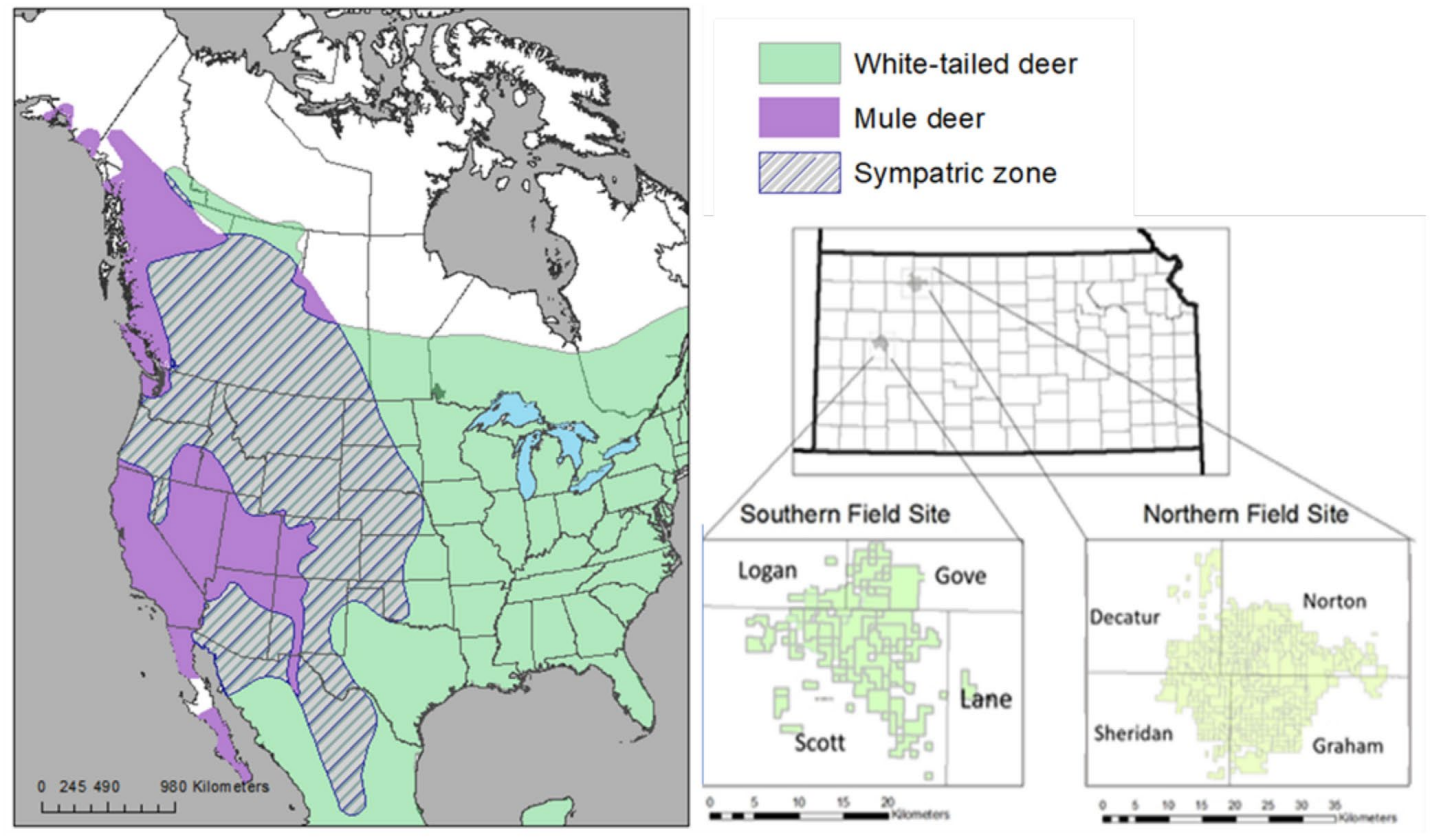
Map depicting range of white‐tailed deer and mule deer in North America and area of sympatry. Study sites within the state of Kansas are separated into North and South populations, and sampling areas span four counties within each site

White‐tailed deer (*Odocoileus virginianus*; WTD) and mule deer (*O*. *hemionus*; MD), the latter which includes Pacific Northwest black‐tail deer (BTD), are broadly distributed across North America with distributional centers in the east (WTD) and west (MD/BTD), but with broad and spatially complex regions of sympatry through the western Great Plains and Inter‐mountain West (Figure 1). Both WTD and MD have experienced dynamic range change during the last century with a predominantly westward shift reflecting expansion and proliferation of WTD concurrent with contraction and population decline of MD that signal the potential for increased interspecific interactions (Bradley et al., 2003; Cronin, 1991; Hornbeck & Mahoney, 2000). Both MD and WTD exhibit distinct regional diversity across North America resulting in recognition of multiple morphological sub‐species and local ecotypes that may represent evolutionarily significant units (Cronin, 1992; Haines et al., 2019; Latch et al., 2009). As distinct evolutionary units often confer local fitness advantages, including differential resistance or susceptibility to pathogens and disease (Blanchong et al., 2016; Grear et al., 2010), adaptive management should address ongoing local population changes from both ecological and evolutionary perspectives. This is particularly important for economically and culturally influential species such as deer with complex histories of management that include multiple population reintroductions and human‐induced population fluctuations (Dawe & Boutin, 2016; DeYoung et al., 2003; Knoche & Lupi, 2012; McClure et al., 2005; Riley et al., 2003; Sawyer et al., 2017; Vercauteren et al., 2011).

In Kansas, MD and WTD ranges overlap throughout private agricultural, U.S. Department of Agriculture Conservation Reserve Program, riparian, and native mixed‐ and short‐grass prairie land cover types. Kansas Department of Wildlife and Parks (KDWP) is currently implementing field‐based monitoring and sampling of both species to better understand (1) ongoing declines in MD through this region and (2) recent increases in incidence of chronic wasting disease (CWD) among both deer species. Given a relative lack of topography through this region, selected habitats between the deer species are not structured by elevation as in other areas, leading to increased syntopy. Improved understanding of how these species are interacting is an urgent priority within the state. Here, we provide an evolutionary perspective on interactions between two North American deer species of prominent management concern under contemporary global change.

In addition to ecological interactions, MD and WTD have been shown to hybridize where populations overlap. Hybrids of both sexes may be viable and fertile, with hybrids analyzed from a genetic perspective found to consist of multi‐generational backcrossed individuals (Bradley et al., 2003; Cathey et al., 1998; Russell et al., 2019). However, there exists a continuing lack of comprehensive genomic specimen resources housed in research archives for assessing relationships among North American deer, as with most game species, particularly other large mammals (Russell et al., 2019). In addition, there is only sparse evidence of hybridization between MD and WTD based on intermediate morphology within wild populations (Bradley et al., 2003). This has hindered effective investigation of the spatiotemporal history of hybridization and the functional consequences of this for future population viability, considering ongoing demographic trends coupled with rapidly increasing incidence of CWD.

Hybridization is a primary area where genomic analyses have advanced our understanding of evolutionary interactions among wildlife species (Boivin et al., 2016; Crispo et al., 2011; McFarlane & Pemberton, 2019). Interspecific hybridization has been well documented but originally was not considered common between native wildlife species (Rhymer & Simberloff, 1996). Hybridization from the perspective of gene‐pool mixing among species is a continuing challenge for developing wildlife policy, particularly for the conservation of rare or declining wildlife that exhibit legacies of, or continued, gene flow (Chan et al., 2019; Haig & Allendorf, 2006). As such, a greater understanding of the evolutionary consequences of hybridization is vital as pertaining to the future integrity of parental species and toward recognition of quantifiable units of management along the speciation continuum (Fitzpatrick et al., 2015). For instance, hybridization may hinder our ability to recognize independent species, and hybridizing species may lose local adaptive advantages through genomic swamping (Allendorf et al., 2001; Hamilton & Miller, 2016; Martinsen et al., 2001; Rhymer & Simberloff, 1996). Given modern genomics, it is increasingly evident that hybridization is relatively common as related taxa differentiate across spatiotemporal axes (e.g., Cetacea, Crossman et al., 2016). Hybridization thus may be considered a potentially pervasive mechanism for promoting divergence between incipient species, and conferring adaptive potential through introduced genetic variation, particularly on the periphery of distributional ranges (Carlson et al., 2014; Meier et al., 2017; Quilodrán et al., 2020; Whiteley et al., 2015).

Introgressive hybridization (IH), where hybrid individuals backcross with one or both of the parental species through successive and continuing generations, is an important concept in wildlife conservation because such continued gene flow makes diagnosis of independently evolving units difficult (Abbott et al., 2013; Chan et al., 2019; Harrison & Larson, 2014). We still know little of functional consequences as IH can result in new genotypes (combinations of gene variants) and also significant shifts in functional genomic variability (Barton & Hewitt, 1985; Schwenk et al., 2008; Shurtliff, 2013). Introgressive hybridization has been demonstrated between multiple species within the family Cervidae in addition to MD and WTD. This includes hybridization between red and sika deer in multiple regions (genus *Cervus*; Goodman et al., 1999, McDevitt et al., 2009, Senn & Pemberton, 2009), sambar and Javan deer (genus *Rusa*; Martins et al., 2018), and chital and hog deer (genus *Axis*; Hill, Linacre, et al., 2019; Hill, Havird, et al., 2019). As such, and given their cultural and economic importance, deer constitute an important focal group for assessing hybrid dynamics.

We applied genomic sequencing methods to a robust sample of sympatric MD and WTD from prairie and agricultural landscapes of the western Great Plains to expand our understanding of gene flow and landscape dynamics for these species. We used reduced representation genomes sampled from free‐ranging MD and WTD across two experimental research populations to provide detailed insight into their spatial population genomic structure. Specifically, we used data from maternally inherited mitochondrial DNA, thousands of neutrally evolving single nucleotide polymorphisms (SNPs), and a reduced dataset of non‐neutral SNPs to (1) investigate further the matrilineal evolutionary history of MD and WTD across North America (including available mitochondrial sequence data from GenBank) from a phylogeographic perspective, including the frequency and dynamics of hybridization considering multiple episodes of mitochondrial capture; (2) investigate population genomic structure, movement dynamics, and genetic diversity of monitored populations within Kansas to characterize their demographic trajectories from a conservation genomics perspective; and (3) assess the dynamics of nuclear gene flow, based on both neutral and non‐neutral loci, between MD and WTD that was detected from our population samples across a zone of sympatry. Given a growing realization that biodiversity interactions at the genomic level (through hybridization) are being exacerbated by global environmental trends, our work provides additional insights for development of adaptive management strategies for deer through the Great Plains, and whether the consequences of hybridization might benefit or impair future population integrity.

## 2 METHODS

### 2.1 Study area and sampling

Tissue samples (blood) from both MD and WTD were collected from live‐captured animals in late winter (Feb–Mar) across two study regions primarily composed of private land located ~130 km apart in the western third of Kansas, during 2018–2020 (Figure 1). The north site (~850 km^2^ centered at 39°36' N, 100°00' W) was in Graham, Norton, Sheridan, and Decatur counties (Figure 1). The south site (~1370 km^2^ centered at 38°42' N, 100°48' W) was in Scott, Logan, Gove, and Lane counties (Figure 1). Both sites were located in the Central Great Plains and High Plains level III ecoregions. Of the eleven physiographic regions in Kansas, all counties included in both study sites were located in the High Plains region; Norton, Graham, Gove, Lane, and Logan counties are also part of the Smoky Hills physiological region (USDA NRCS https://www.nrcs.usda.gov/wps/portal/nrcs/detail/ks/about/?cid=nrcs142p2_033475).

Given the lack of an elevational gradient that typically segregates sympatric populations of MD and WTD, segregation in Kansas is primarily based on differences in habitat selection. Kansas deer habitat is a spatially variable mosaic of woody, grassland, and agricultural vegetation communities whose ability to support deer fluctuates with season, climatic conditions, intensity of land use, cropping patterns, burning regimes, and degree of human disturbance. Comparatively, MD in Kansas are more strongly associated with grassland cover, whereas WTD will prefer woodland cover. Both study sites were a mosaic of cropland and grassland, composed of short‐ and mixed‐grass prairie. The defining difference between the study areas was the presence of the North Fork Solomon River, which crossed the north study area and included floodplain woodlands along riparian areas. The south site included more draws and elevated “chalk rock” areas relative to the north site. Land cover in the north site was 42% cropland, 18% U.S. Department of Agriculture Conservation Reserve Program (CRP) grassland, 18% native pasture, 5% urban, 2% water, and 15% woodland. Land cover in the south site was 71% cropland, 6% CRP, 15% native pasture, 6% urban, and 2% woodland.

Corn (*Zea mays*), wheat (*Triticum aestivum*), and milo (*Sorghum bicolor*) dominated agricultural crops. Other less abundant crops included sunflowers (*Helianthus annus*), alfalfa (*Medicago sativa*), and soybeans (*Glycine max*). Pasture was typically composed of shorter, grazed, native mixed‐grass prairie. Prevalent grasses in the mixed‐grass prairie included little bluestem (*Schizachyrium scoparium*), sideoats grama (*Bouteloua curtipendula*) and blue grama (*B*. *gracilis*). Tall thistle (*Cirsium altissimum*), broom snakeweed (*Gutierrezia sarothrae*), Nuttall's sensitive briar (*Mimosa nuttallii*), and Indian blanket (*Gaillardia pulchella*) were typical forbs, and widespread succulents included soapweed yucca (*Yucca glauca*) and plains prickly pear cactus (*Opuntia macrorhiza*). Tracts of CRP included tallgrass prairie species: big bluestem (*Andropogon gerardi*), Indiangrass (*Sorghastrum nutans*), and switchgrass (*Panicum virgatum*) intermixed with forbs such as white prairie clover *(Dalea candida*), Maximillian sunflower (*Helianthus maximiliani*), purple prairie clover (*Dalea purpurea*), and Illinois bundleflower (*Desmanthus illinoensis*). Woodlands were composed primarily of shelterbelts, small groups of clumped trees intermittently strewn throughout pastures and the large riparian area in the north site. Prevailing tree species included American elm (*Ulmus americana*), box elder (*Acer negundo*), green ash (*Fraxinus pennsylvanica*), hackberry (*Celtis occidentalis*), black cherry (*Prunus serotina*), eastern cottonwood (*Populus deltoides*), honey locust (*Gleditsia triacanthos*), black locust (*Robinia pseudoacacia*), mulberry (*Morus rubra*), black walnut (*Juglans nigra*), and eastern red cedar (*Juniperus virginiana*). Plum thickets (*Prunus angustifolia*) and smooth sumac (*Rhus glabra*) were common shrubs in both study sites.

Adult deer (older than young‐of‐the‐year fawns; ≥1.5 years) were captured during helicopter sessions in February 2018 and 2019 and March 2020 using a commercial helicopter crew (Quicksilver Air Inc., Colorado Springs, CO, USA). All deer were captured and handled according to guidelines approved by the American Society of Mammalogists (Sikes, 2016), under the Institutional Animal Care and Use Committee at Kansas State University (protocol #3963), and authorized under the KDWP scientific permits (SC‐024‐2018, SC‐015‐2019, SC‐032‐2020). Deer were identified as WTD or MD based on pelage coloration and ear length. WTD had tails that were brown above and white below, while MD tails were completely white except for the black tip (Nowak, 1999). Additionally, deer were identified as MD if the ears were more than half of the length of the head, WTD ears were one‐half of the length of the head or shorter. We captured no deer where the species was unclear based on pelage coloration and ear length. MD antlers branch in forks of two relatively equal tines, while WTD antlers have single tines branching from a main beam (Nowak, 1999). However, at time of sampling, some male deer had already shed antler, making this method of identification inconsistent.

A total of 92 deer yielded blood samples (~5 ml) taken via jugular or cephalic puncture for genetic analysis. For MD, this included 27 individuals from the northern population (20 female, 7 male) and 21 from the southern population (12 female, 9 male). For WTD, samples included 24 individuals from the northern population (16 female, 8 male) and 18 from the southern population (10 female, 7 male; Table S1). Additionally, three WTD from outside the study region were sampled (including 1 male from Greenwood County, KS, and 2 females from Trempealeau County, WI) as extralimital samples.

### 2.2 Mitochondrial cytochrome b sequencing and analyses

#### 2.2.1 Samples and Sequencing

Genomic DNA was extracted from 100 µl of blood for each sample following the NEB Monarch Blood DNA extraction kit using manufacturer's instructions. The full mitochondrial cytochrome b (Cytb) gene was amplified for a random subset of deer specimens, including MD (*n* = 31), WTD (*n* = 31), and most of the putative hybrids (*n* = 7) identified from ddRADseq Structure analyses, using primers Odh‐cytbF‐14153 (5′‐TCAATGACCAACATCCGAAA‐3') and Odh‐cytbR‐15399 (5′‐GGGTGTTGATAGTGGGGCTA‐3′), with PCR conditions similar to those of Latch et al. (2009). All PCR products were confirmed on a 2% electrophoresis gel and sequenced in both directions (on an ABI 3730) at Genewiz LLC. Sequences were reconciled in MEGA 10.0 (Tamura et al., 2013). The final data set consisted of sequences trimmed to 1103 bp in length. All sequences were deposited in GenBank (Table S1). For phylogeny reconstruction, we downloaded all additional available Cytb sequence data from GenBank >400 bp in length (*n* = 89), for both BTD (*n* = 17), and WTD (*n* = 153) (Table S1).

#### 2.2.2 Gene tree

We estimated an independent genealogy for the Cytb locus including incomplete sequences and without assigning haplotypes (*n* = 328). We produced chronograms through Markov chain Monte Carlo (MCMC) searches in BEAST2 (Bouckaert et al., 2014), setting all parameters in BEAUti, part of the BEAST2 software package, and estimating the substitution model through use of the bModelTest package, also in BEAST2 (Bouckaert & Drummond, 2017). We applied a relaxed clock: uncorrelated log‐normal molecular clock model and set the mutation rate to 0.045 (4.5% per million years; Latch et al., 2009). We used empirical base frequencies, and a constant population size tree prior, with other parameters run with default settings. We ran MCMC for 50 million generations, sampling every 1000 generations, with the first 1000 trees discarded as burn‐in. Stationarity of MCMC runs was assessed in Tracer v1.7 (Rambaut et al., 2018). We annotated tree files in TreeAnnotator (BEAST2 package). Chronograms were visualized with posterior probabilities in FigTree v1.3.1, and reported as an unrooted tree for ease of interpretation following retrieval of node coalescence times.

#### 2.2.3 Population demographics

Cytb genetic diversity and demographic analyses were performed considering only sequences for deer sampled from the focal study areas in western Kansas. Based on comparison of clade placement retrieved from the Cytb genealogy and morphological species assignment, all WTD were grouped in a single clade but mitochondrial capture of some WTD haplotypes by MD was evident, resulting in two mitogroups for MD. For diversity and demographic analyses, we therefore grouped deer as WTD_All, MD_Mule‐Clade, and MD_White‐tail‐Clade. For each group, we calculated summary statistics and assessed number of haplotypes (*h*), haplotype diversity (*Hd*), nucleotide diversity (*π*), and pairwise sequence divergence in DnaSP v5 (Librado & Rozas, 2009). For tests of demographic expansion, we used DnaSP to calculate Tajima's *D* (Tajima, 1989) and Ramos‐Onsins and Rozas R2 (Ramos‐Onsins & Rozas, 2002). We assessed significance with 10,000 coalescent simulations.

### 2.3 Genomic sequencing and analyses

#### 2.3.1 SNPs

DNA was quantified using Quant‐iT Picogreen dsDNA Assay (Invitrogen) and visualized using gel electrophoresis (2% agarose). Samples all had sufficient yields of high molecular weight DNA (>100 ng) and were submitted to the University of Minnesota Genomics Center (UMGC), Minneapolis for ddRADseq amplification and sequencing. Following an in silico digest of the reference genome, it was determined which restriction enzymes were optimal using a sub‐sample of 8 individuals. UMGC prepared ddRADseq libraries and sequenced samples using the following protocols. For each sample, 100 ng of DNA was digested with 10 units each of *Sbf*I and *Taq*I restriction enzymes from New England Biolabs (NEB) and incubated at 37°C for 2 h before heat inactivating at 80°C for 20 min. Samples were then ligated with 200 units of T4 ligase (NEB) and phased adaptors with CRYG and CG overhangs at 22°C for 1 h before heat killing. The ligated samples were purified with SPRI beads and then amplified for 18 cycles with 2× NEB Taq Master Mix to add unique barcodes to each sample. Libraries were purified, quantified, pooled, and size selected for the 300–744 bp library region and diluted to 2 nM prior to sequencing. UMGC sequenced 150‐bp single‐end reads across 0.25 lanes of a NextSeq 550 High‐Output FlowCell (Illumina, USA). The resulting fastq files were demultiplexed using Illumina bcl2fastq software and Trimmomatic (Bolger et al., 2014) was used to remove adapter sequences (the first 12 bases) from the 3’ ends of reads.

#### 2.3.2 Data filtering

Raw Illumina reads were inspected with FASTQC software (available at http://www.bioinformatics.babraham.ac.uk/projects/fastqc). To process ddRADseq data and produce single nucleotide polymorphism (SNP) data sets, we used the *process_radtags* module of STACKS 2.5 (Rochette et al., 2019) to filter out low‐quality reads, demultiplexing individuals into their own fastq file. We aligned reads from each individual to a WTD reference genome assembly (Ovir.te_1.0; GenBank assembly accession: GCA_002102435.1) using BWA‐MEM algorithm of the Burrows‐Wheel‐Alignment tool v0.7.17 (Li, 2013) with default settings. Previous studies have found high mapping rate (75%) when aligning MD to the WTD reference genome (Russell et al., 2019). Mapped reads were sorted and indexed using SAMtools (Li et al., 2009). We then ran *ref_map* and *populations* pipelines within STACKS, retaining loci found in at least 80% of samples (*r* = 0.80), with a minor allele frequency of at least 5% (min_maf = 0.05), and heterozygosity upper bound of 0.8 (max_het = 0.8) that produced a variant call format (VCF) file. We also only retained one single nucleotide polymorphism (SNP) per locus (‐‐write_single_snp), to meet the assumptions of linkage equilibrium in subsequent analyses. The VCF file was filtered using VCFtools (Danecek et al., 2011) to only include reads with a minimum read depth of 20 and keep individuals with less than 30% missing data. We then used this filtered VCF file for all subsequent analyses.

#### 2.3.3 Genetic diversity and relatedness

Numbers of alleles (*N_A_*), effective numbers of alleles (*N_E_*), expected (*H_E_*), and observed (*H_O_*) heterozygosity, and inbreeding coefficients (*F*_IS_) were calculated for each sampled population in STACKS software using the *populations* module. Estimates of pairwise *F*_ST_ and *D*_Jost_ were calculated in R packages “*hierfstat*” (Goudet, 2005) “*mmod*” (Winter, 2012) in order to understand the degree of variation attributable to putative population structure. Estimates of gene flow and directionality in units of per generation × μ were calculated using Migrate‐N v3.6.11 (Beerli, 2006). This software estimates gene flow for multiple populations, interpreting all shared polymorphism results. We ran the model for all possible connections between population pairs, after uniform priors were determined, we ran one long chain with 10,000,000 parameter values and a burn‐in of 50,000, sampling parameter values every 100 steps.

#### 2.3.4 Identification of outlier loci under selection

To examine the influence of selection on estimates of differentiation and diversity with our ddRADseq dataset, we employed two statistical tools for the identification of putative outlier loci, BayeScan v2.1 (Foll & Gaggiotti, 2008) and *pcadapt* v4.1.0 (Luu et al., 2017). For these analyses, we included all deer samples from both species and populations in western Kansas, but excluded the extralimital WTD to ensure our analysis focused on identifying outlier loci associated with local populations within the sympatric zone. We used BayeScan for estimating the posterior probability that a given locus is affected by selection. Briefly, prior odds of 10 (prior belief that a selection model is 1/10 as likely as a neutral model for a given SNP), 100, and 1000 were used for identifying the top candidates of the selected loci and a total of 50,000 reversible‐jump Markov chain Monte Carlo chains were run with a thinning interval of 10, following 20 pilot runs of 5000 iterations each and a burn‐in length of 50,000. An R function “plot_R,” provided along with the BayeScan software package, was used to plot and identify outliers using different criteria from the BayeScan output file. BayeScan analysis identified 59 SNPs as non‐neutral with an interpretation of possible purifying selection, given the very low *F*_ST_ values of outliers (Figure S1). No outlier loci exhibited a signal of positive selection.

Recent development of multivariate methods such as *pcadapt* allows the identification of outlier loci in admixed or continuous populations. For this analysis, individuals are not sorted into predefined populations. Instead, *pcadapt* ascertains population structure using principal component analysis (PCA) and then identifies markers under putative selection as those that are excessively correlated with population structure. A scree plot of the first 20 principal components (termed *K* in *pcadapt*) indicated that the optimal *K* from our data was 2 for computing correlations between loci and *K* principal components. We used Benjamini and Hochberg's (1995) method for correction of the false discovery rate in both BayeScan and *pcadapt* at *α* = 0.05. Based on the results of both analyses, we separated our ddRADseq data into neutral loci and non‐neutral loci, using a custom script (https://github.com/fraser‐combe). This uses VCFtools to create separate VCF files for neutral and non‐neutral locus sets. For non‐neutral loci, we conservatively included only loci found to be under selection from both outlier methods for further analyses and excluded any loci recovered from only a single method from further analyses. *pcadapt* analysis identified 1580 SNPs (optimal *K* = 2 according to a scree plot; Figure S2) as non‐neutral outliers based on *F_ST_* between WTD and MD. Twenty‐nine outliers were common between *pcadapt* and BayeScan. As such, for subsequent analyses we used 1580 non‐neutral loci recovered from one or both methods, and the remaining 23,438 loci constituted our neutral dataset. These datasets were analyzed separately as indicated by section.

#### 2.3.5 Genetic clustering using multivariate and Bayesian approaches

Discriminant analysis of principal components (DAPC) is a multivariate approach that performs a principal components analysis (PCA) in a first step and then subjects the PC scores to a discriminant function analysis (DFA). Unlike PCA, DFA fits orthogonal discriminant functions that maximize variation between‐group relative to within‐group, making it well suited to differentiating genetic groups (Jombart, 2008). A K‐means clustering approach can be applied to assess the number and composition of K genetic clusters in the data. The best supported model is identified using the Bayesian information criterion (BIC), where the lowest BIC, which is often indicated by an elbow in the curve, is preferred. We performed PCA and DAPC in the “adegenet” package (Jombart, 2008) in R (R 3.6.3, R Core Team, 2020) using the neutral loci only in order to eliminate the possibility of selection influencing cluster assignment. The optimal number of PCs to be retained was determined using the a.optim.score function with 10,000 simulations for each number of PCs retained. In both analyses, we retained the first 10 PCs, which explained 82% of the total variance, retaining all the discriminant functions.

We investigated the presence of population structure and hybridization by analyzing the neutral SNP dataset in the software program STRUCTURE version 2.3.4 (Pritchard et al., 2000). In STRUCTURE, a Bayesian algorithm was used to assign individuals to a value of K clusters. The likelihood that a given individual belongs to a particular cluster is given by a *Q*‐value. Higher *Q*‐values indicate a greater posterior probability that an individual belongs to that cluster. All other individuals were considered hybrids (Juha‐Pekka & Primmer, 2005). We executed runs with a burn‐in of 100,000 iterations followed by 1,000,000 iterations and performed 10 replicate runs for *K* = 1 through *K* = 10. For the STRUCTURE analyses, we set the parameters to allow for admixture between clusters and selected the correlated allele frequency model. The likely number of genetic clusters (*K*) was selected by evaluating mean likelihood scores and ∆*K* implemented in Structure Harvester (Earl & VonHoldt, 2012; Evanno et al., 2005) and Bayesian information criterion (BIC) plots in R package Adegenet (Jombart, 2008).

#### 2.3.6 Hybridization

All hybridization analyses were performed using the neutral dataset except for phylogenetic reconstruction in which both neutral and non‐neutral data were analyzed separately. We compared genomic hybrid indices with interspecific heterozygosity to determine whether individual deer were recent‐generation hybrids (first generation—FI, or second generation—F2) or descendants of extended backcrossing (Bouchemousse et al., 2016). We designated individuals as parental WTD or MD if their respective ancestry proportions exceeded 0.98 in our combined *K* = 2 STRUCTURE runs (Scordato et al., 2017) and individuals with mixed ancestry were assigned as putative hybrids with values of *Q* > 0.05 or <0.95. For each individual, we calculated the genomic hybrid index (*HI*) and the average interspecific heterozygosity across loci using the R package INTROGRESS version 1.2.3 (Gompert & Buerkle, 2010). Parental WTD were set to have a *HI* of 1, and MD were set to a *HI* of 0. The proportion of loci in each admixed genome with alleles inherited from each parental species (i.e., interspecific heterozygosity) was calculated. This method for calculating interspecific heterozygosity assumes that parental allele frequencies are known. Therefore, the same individuals used as parentals for *HI* estimation were also used to calculate interspecific heterozygosity. A triangle plot is used to represent the relationship between the *HI* and interspecific heterozygosity (Fitzpatrick, 2012). F1 hybrids have an expected *HI* of 0.5 and interspecific heterozygosity of 1.0 for loci that are fixed in parental individuals. Heterozygosity is reduced in later‐generation hybrids and backcrosses. We considered deer with *HI* > 0.25 and <0.75, and heterozygosity >0.5 to be recent‐generation hybrids, individuals with *HI* > 0.25 and <0.75 but with heterozygosity <0.5 to be later‐generation hybrids, and deer with *HI* < 0.25 or >0.75 to be multi‐generational backcrosses (Larson et al., 2014; Milne & Abbott, 2008; Scordato et al., 2017).

We also tested for introgression using the ABBA‐BABA test (Durand et al., 2011; Martin et al., 2015). The ABBA‐BABA test (also called Patterson's *D* statistic) can be performed on any four‐taxon tree in the form (((P1, P2), P3), outgroup) to identify introgression between P3 and either P1 or P2. Using our SNP data set, we used DSUITE, a tool that calculates *D* statistics and *f_4_* ratios directly from VCF files created from STACKS analyses, and assessed significance using jackknifing (Malinsky et al., 2021). Under this framework, we calculated D for all combinations of MD and WTD (north and south), assessing introgression between P3 and P2 with the outgroup being the extralimital WTD.

Population tree topology was estimated using the maximum‐likelihood (ML) method implemented in TreeMix (Pickrell & Pritchard, 2012). TreeMix models relative genetic drift at genome‐wide SNP polymorphisms to infer relationships between populations. It first estimates a dendrogram of the relationships between sampled populations. Next, it compares the covariance structure modeled by this dendrogram to the observed covariance between populations. When populations are more closely related than modeled by a bifurcating tree, it suggests that there has been admixture in the history of those populations. TreeMix then adds an edge to the phylogeny, resulting in a phylogenetic network indicating the direction and intensity of gene flow. The position and direction of these edges are informative; if an edge originates more basally in the phylogenetic network, it indicates that this admixture occurred earlier in time or from a more divergent population. We first inferred the ML tree using extralimital WTD samples as an outgroup, tested trees for one to six migration events, with 10 iterations of each, with blocks of 1000 SNPs to account for linkage disequilibrium. We plotted the resulting trees and the residual plots in R using the package Pot (Fitak, 2021) to calculate the second‐order rate of change in the log‐likelihood to infer the number of migration events.

We also generated a phylogenetic network analysis using SplitsTree4 version 4.14.6 (Huson & Bryant, 2006), which takes account of more realistic models, such as loss and duplication events, hybridization, or recombination. We used Phylip files based on ddRADseq data, and within the program, we used uncorrected p‐distances, NeighborNet, and then EqualAngle to compute an unrooted network for all populations/individuals. In order to test each split, our matrix was bootstrapped with 100 replicates using default parameters.

#### 2.3.7 Inferred demographic histories

The demographic histories of our species/populations were inferred based on neutral loci using the STAIRWAY PLOT V2 software (Liu & Fu, 2015). This method has a demonstrated utility for ddRADseq data and considering effective population size (*N_e_*) changes through the evolutionary time frames of our study species. We set the mutation rate to 1.1 × 10^−8^ and generation time to 5 years as used previously in cervids (Jong et al., 2020). We generated species‐specific folded site frequency spectrum (SFS) vectors with a custom‐built script, from which we then calculated the size of each bin (i.e., number of SNPs within each bin) for input into the software.

## 3 RESULTS

### 3.1 Mitochondrial DNA

**FIGURE 2:**
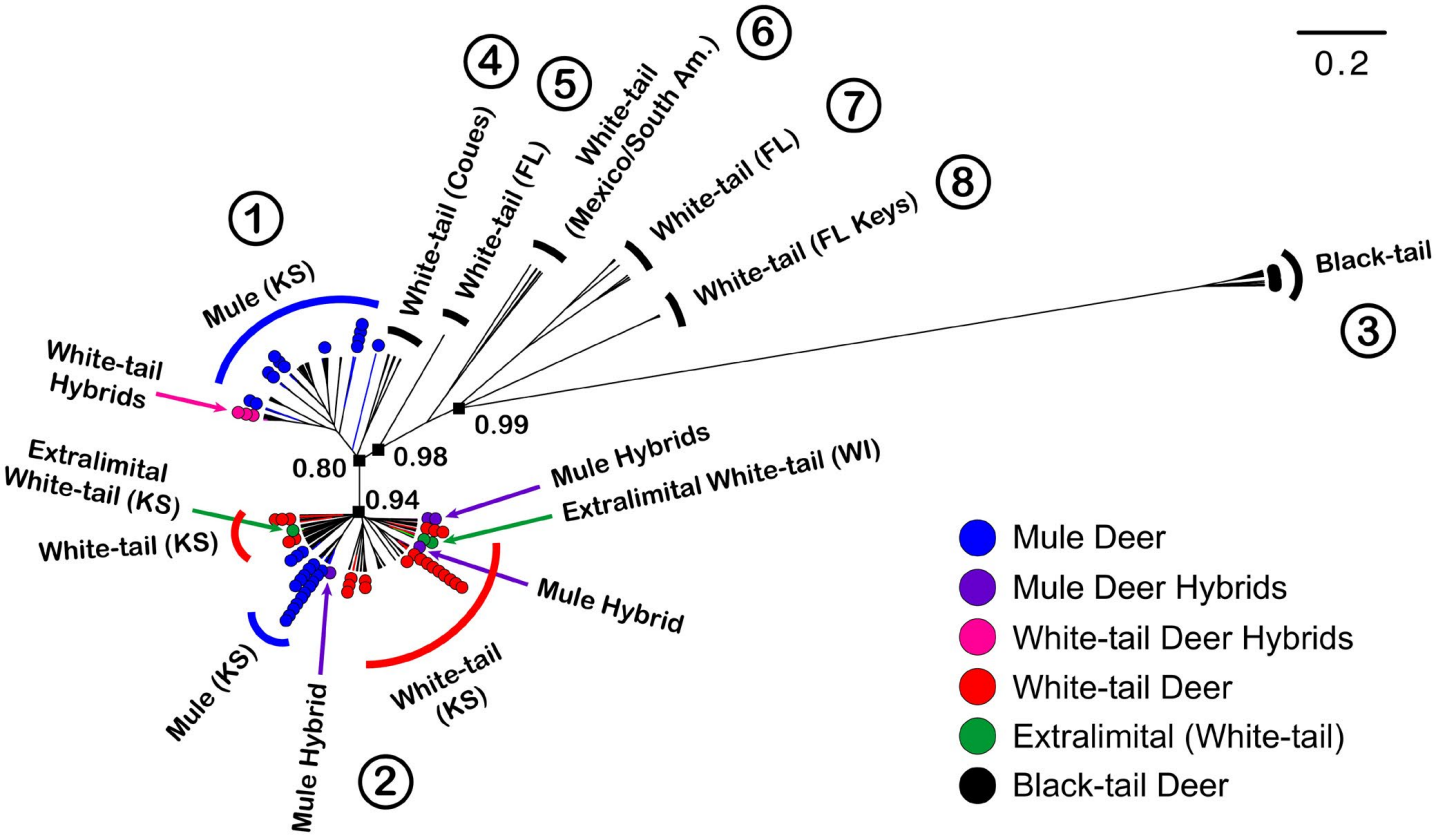
Bayesian phylogeny for *Odocoileus* spp. based on the mitochondrial cytochrome b gene (1103 bp). Posterior probabilities for major nodes are illustrated; numbers represent clade designations. Each dot and associated branch represents a sample contributed from this study

The Cytb genealogy reflects a complex history of diversification among *Odocoileus* deer that includes multiple episodes of mitochondrial capture through interspecific gene flow (Figure 2; Table S1). The matrilineal relationships among MD, BTD, and WTD indicate that all MD and WTD form a well‐supported and reciprocally monophyletic group with respect to BTD (Figure 2 – Clade 3). Within the MD/WTD clade, WTD from Florida (Clades 5, 7), Florida Keys (Clade 8) and from Mexico and South America (Clade 6) form multiple distinct genetic clades that are divergent from all other deer. Coues WTD (Clade 4) form a moderately well‐supported relationship with most MD samples (Clade 1) and including the hybrid individuals from our study that are morphologically assigned to WTD. A separate well‐supported clade (Clade 2) contains most WTD (including our extralimital samples), some MD, and hybrid individuals from our study that are morphologically assigned to MD (Figure 2; Table S1). Coalescence times for all clades are within the timeframe of the Quaternary Period with all deer samples coalescing at ~1.2 Ma and all MD (excluding BTD) and WTD coalescing at ~0.5 Mya (Table 1). The coalescent history of the predominantly MD clade (Clade 1) is dated to ~190 kya, and the predominantly WTD clade (Clade 2) is dated to ~140 kya. Given the mtDNA genealogical clade assignments of deer from the Kansas study regions and given also that there was no mtDNA structure by geography between the north and south study regions, we considered four groups for genetic diversity and demographic statistics that also reflect matrilineal history (Table 2). All deer combined exhibited high haplotype diversity and moderately high nucleotide diversity. Combined WTD_All from Kansas exhibited high haplotype diversity but relatively low nucleotide diversity. MD_White‐tail‐Clade (from Clade 2) exhibited very low nucleotide diversity. Conversely, MD_Mule‐Clade (from Clade 1) exhibited a haplotype diversity of 1.0 and highest values of nucleotide diversity, but this was accompanied by a positive value for Tajima's *D* (Table 2). No groups displayed consistent significant signals of recent demographic expansion.

**TABLE 1:** Coalescence times (Myr) for mtDNA clades (including 95% confidence interval) based on an estimated mutation rate of 4.5%/Myr for mule deer, white‐tailed deer and black‐tailed deer

Group	Coalescence times
Black‐tail (3) – Mule/White‐tail (1, 2, 4–8)	1.20 (0.65, 1.84)
White‐tail (FL Keys; 8) – All other Mule/White‐tail (1, 2, 4–7)	0.51 (0.36, 0.69)
White‐tail (MX, S. Am.; 6) – All other Mule/White‐tail (1, 2, 4, 5, 7)	0.42 (0.29, 0.56)
Ingroup (All other Mule/White‐tail; 1, 2, 4)	0.25 (0.18, 0.33)
White‐tail (Coues; 4) – Mule (1)	0.22 (0.15, 0.29)
Mule (1)	0.19 (0.13, 0.27)
All other Mule/White‐tail (2)	0.14 (0.09, 0.19)

Note

Clades are reported both by taxon/geography and by clade numbers as reported in Figure 2.

**TABLE 2:** Genetic diversity statistics and tests of demographic expansion for deer populations in western Kansas

Group	*N*	*h*	*S*	*Hd*	*π*	*D*	*R2*
All	66	54	57	0.990	0.0079	−0.909 (0.202)	−0.073 (0.176)
WTD_All	28	23	29	0.979	0.0047	−1.139 (0.119)	0.079 (0.080)
MD_Mule‐Clade	13	13	31	1.000	0.0095	0.219 (0.664)	0.073 (0.195)
MD_White‐tail‐Clade	18	14	12	0.967	0.0024	−0.870 (0.195)	**0.096 (0.050)**

Note

Populations reflect morphological species designation and clade membership as follows: All = all mule and white‐tail deer from study area; WTD_All = all white‐tail deer from study area; MD_Mule‐Clade = all mule deer within the predominantly mule deer clade (Clade 1 from Figure 2); MD_White‐tail‐Clade = all mule deer exhibiting mitochondrial capture within the predominantly white‐tail clade (Clade 2 from Figure 2). Hybrid deer were not included. Statistics include the following: *n*, sample size; *h*, number of haplotypes; *S*, number of segregating sites; *Hd*, haplotype diversity; *π*, nucleotide diversity; *D*, Tajima's *D* (including *p*‐value); *R2*, Ramos‐Onsins and Rozas *R2* (including *p*‐value). Bold value is significant at *p* = .05.

### 3.2 Genomic sequencing

We recovered 98,272,337 high‐quality reads (*Q* > 30) across 94 samples (mean = 1,045,450 reads). After filtering for read quality and presence of correct barcodes, *Sbf*I and *Taq*I recognition sites, a total of 78,496,017 read pairs were generated across all samples. After all quality filters that met our coverage and missing data criteria, and condensing our data to one SNP per loci, a total of 25,018 polymorphic SNP loci with high read depth coverage (33.45–90.35) were retained for further analysis of genetic diversity, population structure, and hybridization. No individuals were removed after filtering.

#### 3.2.1 Genetic diversity and connectivity

All measures of genetic diversity from nuclear data, including *H_O_*, *H_E_*, and *π*, were greater for WTD than MD (Table 3). MD from the northern population had greater diversity compared to the southern population, whereas estimates for WTD (including extralimital samples) were relatively similar between sites. There were greater numbers of private alleles for WTD than MD; northern populations had greater numbers of private alleles than southern populations for both species. Similar values of *H_O_* and *H_E_* coupled with relatively low *F_IS_* values suggest low levels of inbreeding, although highest *F_IS_* values for northern MD and northern WTD may reflect non‐random mating in these populations (Table 3). All estimates of pairwise *F_ST_* indicate high connectivity between northern and southern populations, for both MD (*F*_ST_ = 0.02) and WTD (*F*_ST_ = 0.014; Table 4). Values of *F*_ST_ between MD and WTD among study populations were roughly twice as low as between MD and extralimital WTD, possibly reflecting a lack of reproductive isolation between species within the study region. WTD from the study region and extralimital WTD were genetically very similar (*F*_ST_ = 0.025–0.032). Estimates of directional gene flow from northern to southern populations of MD were high, corresponding to 20 (95% confidence interval [CI, 18.4–21.2]) migrants per generation, but were low when considering dispersal from southern to northern populations, at 4 (CI, 0.36–0.45) migrants per generation. For WTD estimates of dispersal in both directions were ~15 (CI, 14.2–16.3) migrants per generation, indicating no barriers to gene flow between these populations.

**TABLE 3:** Genome‐wide population summary statistics. Included are sample size (*n*), number of polymorphic sites (*N*_sites_), private alleles (*A*_private_), observed and expected heterozygosity (*H_O_*, *H_E_*), nucleotide diversity (*π*) and inbreeding coefficient (*F*_IS_)

Species	Population	*n*	*N*_sites_	*A*_private_	*H_O_*	*H_E_*	*π*	*F*_IS_
MD	North	28	13417	1441	0.060	0.093	0.0945	0.202
MD	South	21	5239	510	0.054	0.054	0.0554	0.008
WTD	North	24	17939	2458	0.112	0.161	0.1646	0.203
WTD	South	18	16757	1776	0.110	0.158	0.1633	0.191
WTD	Extralimital	3	6473	431	0.121	0.116	0.1390	0.035

**TABLE 4:** *F*_ST_ (top‐right) and *D*_Jost_ (lower‐left) values among white‐tailed deer and mule deer from the Kansas study site. Populations are considered as North (N), South (S), and extralimital (ex)

	MD‐N	MD‐S	WTD‐N	WTD‐S	WTD‐ex
MD‐N	–	0.013	0.132	0.134	0.400
MD‐S	0.001	–	0.172	0.184	0.424
WTD‐N	0.070	0.070	–	0.014	0.025
WTD‐S	0.071	0.070	0.003	–	0.032
WTD‐ex	0.084	0.103	0.005	0.005	–

#### 3.2.2 Population genetic structure

**FIGURE 3:**
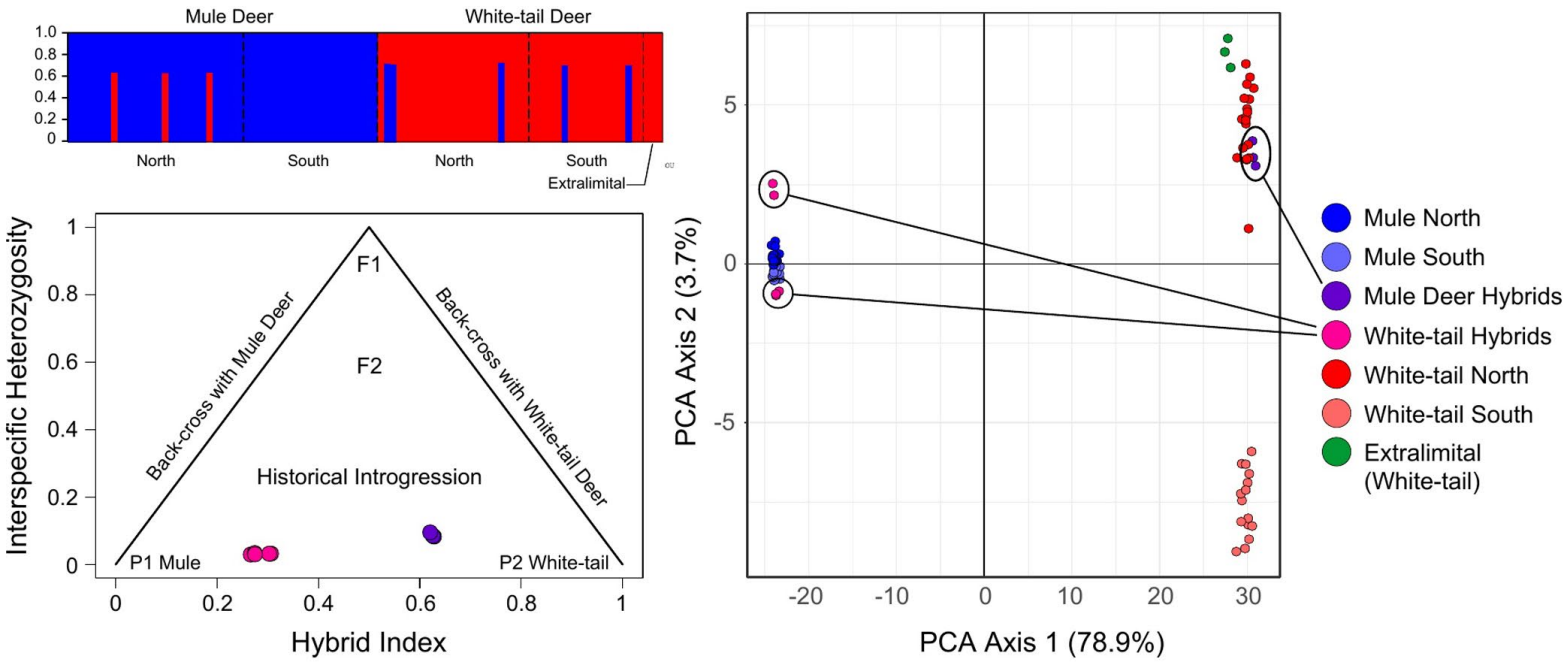
Population genomic structure of white‐tailed deer (WTD) and mule deer (MD) in Kansas. Top‐left: STRUCTURE plot for MD (blue) and WTD (red) assigned by species and geographic population, with putative hybrids identified from admixed q‐scores. Bottom‐left: Triangle plot of interspecific heterozygosity and hybrid index across all SNPs, with P1 and P2 representing non‐hybrid MD and WTD, respectively, F1, First filial; F2, second filial; and Bx, backcross. Hybrid individuals morphologically identified as MD are pink. Hybrids morphologically identified as WTD are purple. Right: DAPC plot based on 23,438 SNPs shared between all populations

Based on multiple clustering methods using SNP data, cross‐validation for all samples and for each subpopulation revealed the optimal number of clusters as *K* = 2, separating MD and WTD by species for both DAPC (through BIC plot) and STRUCTURE (through Evanno method testing *K* = 1–10) analyses. There was no substructure based on location and extralimital WTD were grouped with Kansas WTD samples (Figure 3). DAPC analyses indicated strong support for two clusters corresponding to WTD and MD along the first PCA axis. The second PCA axis again indicated no substructure within MD but existing substructure between northern and southern populations of WTD. The variation explained by the first two principal components was 78.86% for PC1 and 3.65% for PC2 (Figure 3).

#### 3.2.3 Hybridization

**FIGURE 4:**
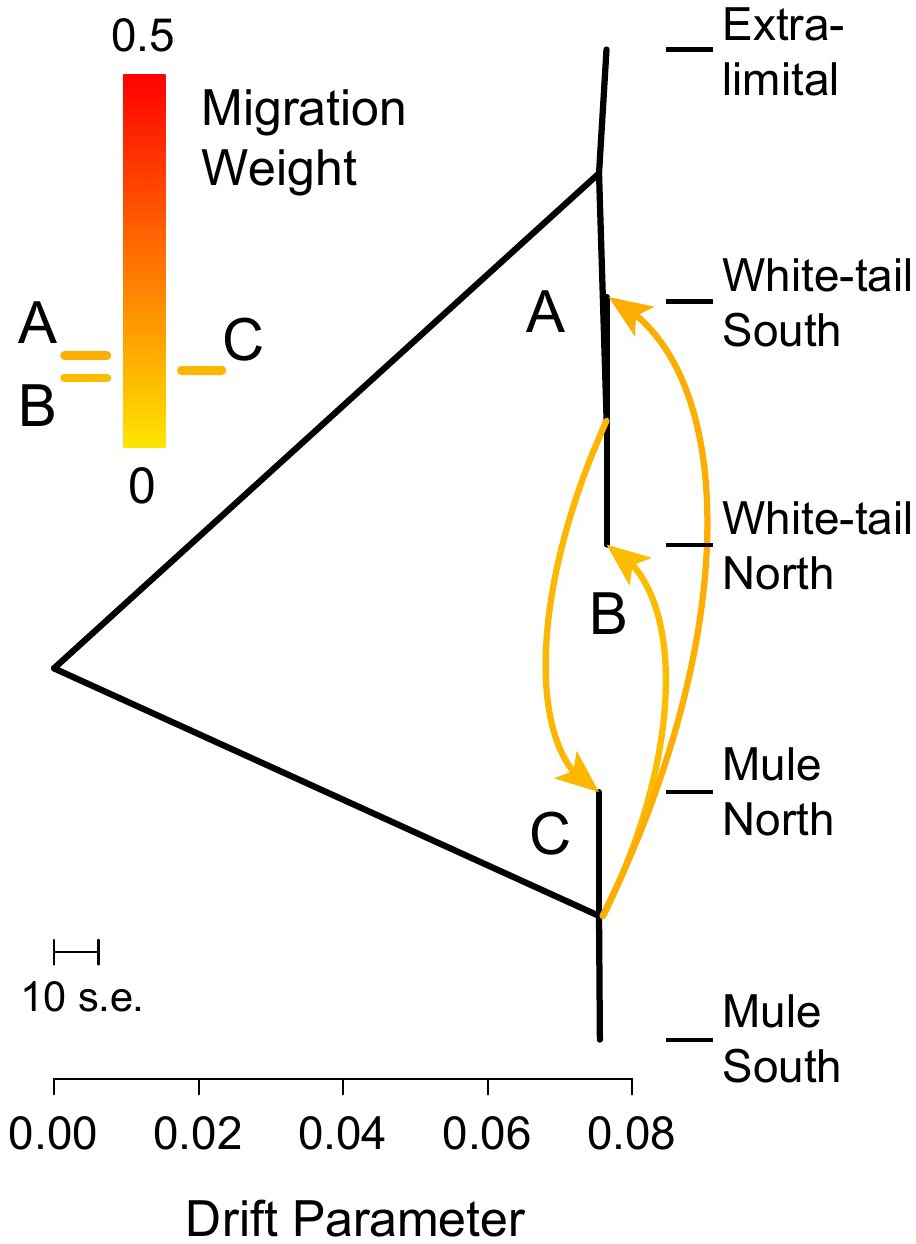
Maximum‐likelihood tree topology with three inferred episodes of gene flow (arrows) with associated weight inferred by Treemix between each population/species, with the tree depth reflecting the drift parameter

Eight putative hybrid individuals were evident across both STRUCTURE and DAPC analyses, where individuals identified as hybrids based on STRUCTURE analysis were spatially offset from non‐hybrid DAPC clusters. Hybrids were identified from the northern MD population (*n* = 3) and from both northern (*n* = 3) and southern (*n* = 2) WTD populations, but no hybrids were identified in the southern MD population. In the DAPC plot, hybrids are not spatially intermediate to WTD and MD but rather group on the periphery of each species cluster. An evaluation of all putative hybrids by INTROGRESS using a triangle plot yielded intermediate *HI* values, consistent with q‐values in STRUCTURE, and coupled with low interspecific heterozygosity. Together, all analyses of hybridization are consistent with backcrossing and historical bidirectional introgression (Figure 3). Values of *HI* ranged from 0.28–0.32 in WTD and 0.62–0.64 in MD and interspecific heterozygosity ranged from 0.04–0.12. For all clustering analyses, hybrid individuals with a predominant assignment of loci to MD had WTD external morphology, and vice versa, hybrids with a predominant assignment of loci to WTD had MD external morphology. Patterson's *D* statistics directly test for introgression using ABBA‐BABA comparisons for all population pairs. We identified significant and positive *D* statistics and related estimates of admixture fraction (*f_4_*‐ratio) in all tests (Table 5), again supporting introgression between MD and WTD (*D* = 0.16–0.26, *p* < .001). From the Treemix analysis, the best fit to the data was obtained with three “migration” events (*p* < .001) indicating gene flow from MD (central node) into both northern and southern WTD populations and also from WTD (central node) into northern MD, providing further evidence of bidirectional gene flow and introgression (Figure 4).

**TABLE 5:** Results for *D* statistic and *f4*‐ratio tests of admixture between white‐tailed deer (WTD) and mule deer (all comparisons) and counts of the BBAA, ABBA, and BABA, patterns. P1, P2, and P3 reflect hierarchical populations used for the ABBA‐BABA test according to (((P1, P2) P3) outgroup) where the outgroup was always the three extralimital WTD samples

P1	P2	P3	*D*	*Z*‐score	*p*‐value	*f4*‐ratio	BBAA	ABBA	BABA
MD‐N	MD‐S	WTD‐N	0.161	21.45	<.001	0.162	2086.8	78.2	56.5
MD‐N	MD‐S	WTD‐S	0.212	33.28	<.001	0.128	2022.9	88.9	57.7
WTD‐S	MD‐N	WTD‐N	0.234	27.36	<.001	0.131	498.1	433.0	268.8
WTD‐S	MD‐S	WTD‐N	0.262	27.23	<.001	0.165	521.5	446.9	261.1

Note

Populations for each species are considered as North (N) and South (S).

#### 3.2.4 Phylogenetic relationships

**FIGURE 5:**
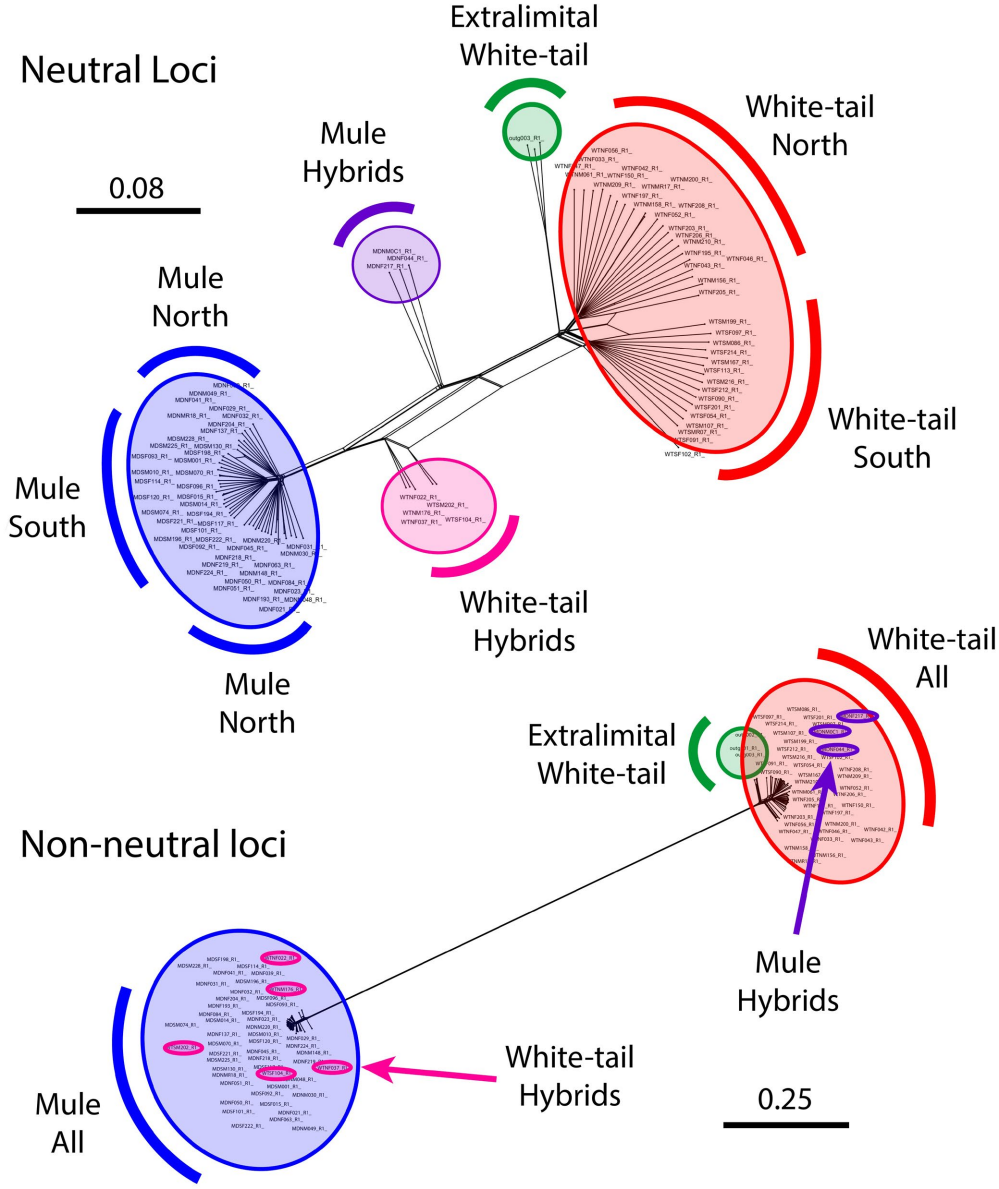
SplitsTree topologies generated by Neighbor‐net and uncorrected *P* distance among white‐tailed deer and mule deer using neutral (non‐outliers) and high *F_ST_* non‐neutral (outlier) SNP data. Top: Tree based on 23,438 putatively neutral SNPs. Bottom: Tree based on 1580 non‐neutral SNPs

Neighbor‐net analyses for neutral SNPs indicate species differentiation between MD and WTD as well as minimal population‐level divergence between northern and southern study regions for both species (Figure 5). Hybrid deer are intermediate reflecting proportions of loci originating from both species. Hybrids designated as MD based on morphology are more genetically similar to WTD, and similarly, hybrids designated as WTD based on morphology are more genetically similar to MD (Figure 5). Higher genetic diversity among WTD is reflected by longer branch lengths in neighbor‐net trees than for MD. Considering non‐neutral loci, only two clusters of individuals were evident, and all hybrids were genetically clustered with the opposite species from their morphological designation (Figure 5).

#### 3.2.5 Demographic history

**FIGURE 6:**
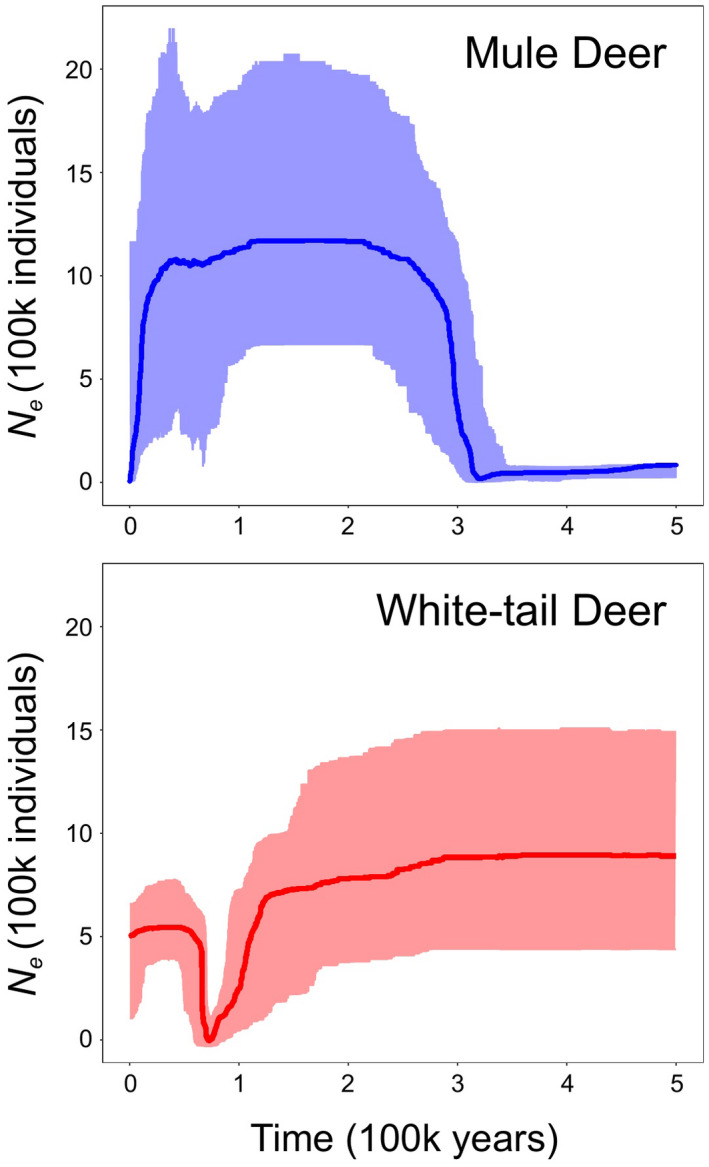
Demographic histories of mule deer (blue) and white‐tailed deer (red) inferred by stairway plot. Thick line = median effective population size (*N_e_*). Shaded areas indicate the [2.5,97.5] percentile intervals

Stairway plots inferring demographic population size change indicated different population histories for MD and WTD consistent with the genomic signals of diversity observed (Figure 6). Both species show evidence of high *N_e_* during the last interglacial period (coincident with ~130 kya). WTD show a significant population decline coincident with onset of the last glacial period (~100 kya) followed by a rapid recovery to contemporary *N_e_* estimates of ~500,000. Conversely, our results suggest that MD had sustained *N_e_* through much of the late Quaternary but have experienced a severe and recent population decline with low contemporary *N_e_* (Figure 6).

## 4 DISCUSSION

We present the first genomic study for deer that develops both a panel of thousands of informative SNPs and a comprehensive assessment of the available mitochondrial Cytb data (the primary haploid locus used for mammals). Our data increase understanding of population genomic structure, quantify the dynamics of hybridization between MD and WTD in sympatry within Kansas, and relate these insights to a long history of genetic interactions among North American deer. Our primary findings include that (1) genetic diversity and demographic trends among deer species in western Kansas are congruent with ongoing ecological population trajectories; (2) given relatively high levels of interbreeding between MD and WTD in western Kansas we recognize yet another region of ongoing hybridization between these species, and this incomplete reproductive isolation reflects genomic introgression in both directions; and (3) mitochondrial capture of WTD haplotypes by MD reflects an ongoing process of interbreeding through the history of diversification of these species. We provide here a discussion of the importance of these findings for managing the future integrity of independent deer species within North America and considering important research priorities for ongoing studies.

### 4.1 Population genetic structure and gene flow

The predominant hypotheses for a contemporary decline of MD, particularly in eastern portions of their range accompanied by expansion of WTD, include changing land use and land cover (farming and ranching practices, woody encroachment, and industrial disturbance), changing climate (long‐term precipitation and temperature regimes), and competitive dominance of WTD over MD (Hornbeck & Mahoney, 2000; Northrup et al., 2016; Sawyer et al., 2017; Walter et al., 2009). While interspecific competition for space and resources coupled with social interactions is currently considered a direct driver of deer population changes, there are several alternative factors related to sympatry that may be detrimental to one or both species, or may confer advantage to one species at the expense of another. Disease can be a primary factor driving population trends through time. Chronic wasting disease within deer and among all North American cervid species is a major and growing concern as incidence of this incurable prion disorder increases, and yet little is known of the genetic basis of susceptibility or of the ecological factors that limit or promote spread of prions through the environment (Escobar et al., 2020; Garlick et al., 2014; Mawdsley, 2020). Additional significant pathogens exist among deer, including brain worm (*Parelaphostrongylus tenuis* nematodes), which generally cause silent infection in WTD but can lead to serious disease in MD (Anderson, 1972). Deer genetic changes associated with population demographic changes may also directly influence population viability. Expanding populations may have increasing abundance accompanied by decreasing genetic diversity along the expansion front (Eckert et al., 2008). Alternatively, declining species and small populations have an increasing likelihood of non‐random mating and subsequent inbreeding depression (Spielman et al., 2004), accompanied by increased genetic drift, both of which can lead to fixation of deleterious genetic variants (Hedrick & Garcia‐Dorado, 2016). Increased interactions among species, particularly when accompanied by pronounced population changes or other stressors may also increase the chance of hybridization between closely related species, with unknown consequences for future fitness (Chan et al., 2019). As such, genomic approaches to population biology can enable important insight toward management priorities.

A first step to investigating these dynamics is to diagnose local population structure, genetic diversity, and movement between populations (Balkenhol et al., 2017; Hohenlohe et al., 2021). We found no population structure between northern and southern study regions based on mitochondrial data and limited but consistent population structure based on neutral SNP data (Figures 3 and 5). In particular, WTD exhibited limited genetic differentiation between study regions based on our DAPC analysis (Figure 3). Given that the westward expansion of WTD in Kansas is a relatively recent phenomenon and that land forms within Kansas are strongly west–east oriented, including Interstate Highway 70 running between northern and southern study sites, it is possible that WTD populations have independently colonized the study regions while maintaining unique identity across one or more barriers (Nixon et al., 2007). Also, given that extralimital WTD samples (from east‐central Kansas and Wisconsin) were likewise genetically divergent from WTD from western Kansas, it is evident that comprehensive SNP data may be informative for recognizing regional genetic populations and potentially for resolving the regional origins of transplanted or dispersing individuals (Chafin et al., 2021).

In this study, regional populations were separated by ~200 km when considering the central point of northern and southern study areas. Elsewhere through North America, migration distances of MD and WTD between winter and summer ranges are known to be as large as 264 km in a single year (Nelson et al., 2004; Sawyer et al., 2016). Our estimated gene flow results do indicate per‐generation bidirectional dispersal by both species between locations, although the direction of gene flow for MD is predominantly from north to south. Greater future sampling will be necessary to more accurately assess landscape connectivity and patterns of gene flow within species. Both species exhibit seasonal migration (although this is less pronounced in Kansas than other regions) and dispersal of individuals from natal areas, and such movements can provide opportunities for outbreeding, significantly changing the genetic composition among populations, and modifying allele frequencies that may counter the effects of both inbreeding and genetic drift (Frankham, 2005; Reed et al., 2016; Storfer, 1999).

Here, we find neutral genomic, and mitochondrial, evidence of relatively low genetic diversity of MD populations in comparison with WTD. This is evident through lower heterozygosity and lower number of private alleles within MD. Mitochondrial genetic diversity is similar to that of other deer populations within North America (Haines et al., 2019). SNP genetic variability was lower in Kansas MD in comparison with a recent genetic assessment of MD in Wyoming (Lacava et al., 2021); however, this comparison should be used cautiously, given differences in sequencing methods. There currently are no comparative SNP data with which to put our WTD populations in a broader context. Although dispersal among populations can be highly beneficial from a genetic standpoint, often as facilitated by humans through conservation‐based transplant and genetic rescue, dispersal may also be detrimental if dispersing individuals carry disease or locally unfit genotypes. Our finding of moderate per‐generation dispersal of both species may therefore have potentially positive or negative implications for deer population trajectories, where movement could minimize effects of inbreeding if that were an issue, but could also introduce pathogens, or minimally changes in susceptibility to existing pathogens (Leiss et al., 2017; Robinson et al., 2012). As such, a more thorough landscape genomics assessment is warranted (Blanchong et al., 2008, 2016).

In addition to genetic diversity and levels of dispersal, we assessed genetic demographics through time, with episodic changes in population trends observed in both species during historical climatic events. Importantly, we found that effective population estimates were generally opposite for the two species, and extremes were temporally congruent, such that when MD populations were low, WTD populations were high, and vice versa. These trends likely reflect changes in both actual population sizes and spatial range extent of the species through time. For WTD, there is a strong signal of a pronounced population bottleneck coincident with onset of the last glacial period. Given this is coupled with a mitochondrial history that suggests persistence of WTD in refugia and associated shifts in geographical distribution through time, we might infer that glacial‐phase populations of WTD were dramatically reduced, followed by a rapid increase in effective population size culminating in current estimates of ~500,000. This is reasonable despite much greater census size estimates for WTD considering highly skewed contributions of males and females to reproduction and also overall low genetic diversity. For MD, high effective population size during the last glacial was followed by a substantial decline roughly coincident with the Holocene warming trend to a very low current level, possibly reflecting significant human‐associated changes in vegetation and land use. This, coupled with a positive value for the Tajima's *D* statistic based on Cytb data, is corroborative of contemporary sudden population decline among MD. These long‐term genetic trends are often attributed to changing climate or other environmental conditions, and as such, we can speculate that current trends in WTD and MD may either directly or indirectly reflect responses to ongoing climate change, likely accompanied by other stressors or environmental factors. Whereas WTD populations are expanding coincident with global warming, MD populations were much larger during colder climates. Similar patterns of recent demographic decline in effective population sizes in other cold‐adapted large mammals based on climatic conditions during this period have been described (Ekblom et al., 2018; Jong et al., 2020; Lucena‐Perez et al., 2020). More comprehensive geographical sampling of deer populations for analysis using methods such as stairway plots would provide greater insight into the demographic consequences of climate variation during the late Quaternary.

### 4.2 Phylogeography and historical gene flow

Given the large number of genetic studies of North American deer, coupled with recognition of ancient hybridization between MD and WTD species, it is surprising that they have not been studied to‐date from a range‐wide and multi‐species comparative perspective. This likely reflects a taxon‐specific or regional focus of previous studies, and a persistent lack of available vouchered genomic resources of deer within museum archives (Cook & Light, 2019; Russell et al., 2019). However, at this time, there exist robust mitochondrial data from multiple independent studies within GenBank that offer greater insight into the evolution of these species from across their combined ranges, although there remain significant portions of their ranges that are not represented in our mitochondrial dataset. Earlier studies have indicated mitochondrial capture through hybridization between MD and WTD (Carr et al., 1986; Cronin et al., 1988). We confirm that here with additional insight to the frequency and timing of these events. All deer (including MD, BTD, and WTD) share a matrilineal origin consistent with a mid‐Quaternary timeframe (1.2 Ma). Although fossils of WTD exist dated to ~3 Ma (coincident with onset of the Quaternary), earliest MD fossils are more recent and consistent with the mid‐ to late Quaternary (Kurtén & Anderson, 1980). Our estimated coalescence time for all deer is also reasonable given that we used a constant population size tree prior for our BEAST analyses although there are considerable population fluctuations within both species through time.

WTD form six well‐supported mitochondrial clades that are geographically discrete and indicate multiple isolated regions within which WTD diverged, across several glacial–interglacial cycles. Haplotypes within multiple Florida clades are genetically distinct from WTD from elsewhere through their North American range, as are haplotypes from Mexico and South America. This suggests three separate population centers existed, in southeast North America, Central America, and likely western North America during one or more glacial periods. The latter area is inferred based on occurrence of MD haplotypes within this WTD clade (Figure 2—combined Clades 1,2, 4) and a coalescence time of 0.25 Ma coincident with the Kansan glacial phase. Considering that all MD Cytb sequences available from throughout their range (*n* = 89) are highly divergent from all BTD, but are closely related to WTD samples, we can infer that MD, and most WTD, were isolated in a single region (separate from BTD) where MD experienced capture of WTD mitochondrial DNA, and suggesting that production of fertile hybrids at this point was between male MD and female WTD. Importantly, inclusion of samples from across the range of WTD indicate that mitochondrial capture by MD occurred subsequent to the divergence of WTD from Florida, Mexico, and South America, further supporting mitochondrial capture as opposed to lineage sorting as an explanation for well‐established mito‐nuclear discordance among these species (Cathey et al., 1998). Subsequently, MD and WTD diverged but again MD experienced capture of WTD mitochondrial haplotypes potentially consistent with the last interglacial–glacial cycle (Sangamon‐Wisconsin).

In terms of breeding dynamics, we know that hybrid individuals may be morphologically assigned to either MD or WTD and may be either males or females. However, we cannot confirm if male or female hybrids contribute differentially to subsequent backcrossed generations. The evidence does suggest that successful hybrids (viable and fertile) during the late Quaternary were the result of interbreeding between male MD and female WTD. However, evidence from other studies (Ballinger et al., 1992; Bradley et al., 2003; Cathey et al., 1998) indicates that modern fertile hybrids may also result from crosses between male WTD and female MD. Based on data from Kansas, no WTD haplotypes group within the predominantly MD clade (Clade 1) whereas a number of MD haplotypes group within the predominantly WTD clade (Clade 2). Accumulating evidence from this and other studies supports uni‐directional ancient hybridization, but potentially bidirectional gene flow in some current geographic areas of hybridization and not others (the latter including existing evidence from the Kansas hybrid zone).

### 4.3 Contemporary hybridization dynamics

Hybridization has been found to be relatively common between ungulate species, in large part due to detection using increasingly complex nuclear datasets, and often in combination with mtDNA that can highlight discordant genealogies, a common signature of interspecific gene flow (Hill, Linacre, et al., 2019; Hill, Havird, et al., 2019; Iacolina et al., 2019). Often, intermediate morphology that may result from initial genetic crossing is not evident within even modestly backcrossed individuals as they assume one or the other parental phenotype. However, it is not clear from our results what genomic mechanism(s) might contribute to the phenotype of hybrids. It would be logical to assume that individuals consistently backcrossing with MD, for instance, would acquire a MD morphology, and yet, our combined data support the opposite finding, where hybrids with MD morphology all have WTD Cytb haplotypes, and a preponderance of WTD neutral SNPs, and vice versa for hybrids with WTD morphology exhibiting a preponderance of MD genetic material. Given the consistency of these results, we hypothesize that some sorting mechanism exists within the exome where critical functional genomic elements must remain true to species assignment through inheritance, and unfit allele combinations as a result of hybridization are purged. If so, this would indicate strong directional selective pressure on hybrids. Although the genes and functions associated with this purifying selection remain unknown, genes associated with phenotype, or which maintain linkage to genes coding for phenotype may well have a role in determining viability of deer hybrids within this system. The most striking example of this mismatch between genetics and morphological assignment is evident from the neighbor‐net tree using the non‐neutral dataset where it appears as if all loci under selection are fixed for the opposite species from morphological assignment. However, this result should be interpreted with caution, given that most non‐neutral loci were recovered using *pcadapt* which explicitly tests for loci that maximize structure between study taxa. Given this skewed result, we repeated the analysis using only the 59 non‐neutral loci from BayeScan analysis and recovered a strikingly similar topology to that for all non‐neutral loci.

We detected eight individuals (8.7%) of hybrid origin, but with clear external morphology of either MD or WTD. All have experienced multi‐generational backcrossing although the number of backcrossed generations is unknown. The consistency of direction of backcross across generations (e.g., always with MD or sometimes with MD and sometimes with WTD) cannot be determined from our data at present. But, given that the proportional assignment of loci to either species (hybrid index) or values for interspecific heterozygosity are very similar among all hybrid individuals as opposed to a more random spread of hybrids across genomic assignment space, functional segregation is likely. Further, patterns of genomic assignment of hybrids are repeated and generally consistent across three groups of hybrids, each with multiple individuals, including two WTD hybrids within MD‐N, three WTD hybrids within MD‐S, and three MD hybrids within WTD‐N. Evidence of balancing selection acting on ungulate species has been identified and may play a role in immunogenetic variation and how species respond to potential diseases and countering the effect of drift (Cavedon et al., 2019; Quéméré et al., 2015; Yao et al., 2015). While balancing selection may be important for maintaining adaptive potential, we found no evidence of excess heterozygosity. Therefore, given all the evidence, the dominant pattern from our data is likely of strong purifying selection for some functional process. For instance, this may be associated with maintaining mito‐nuclear compatibility for vital cellular processes (Hill, Linacre, et al., 2019; Hill, Havird, et al., 2019; Princepe & de Aguiar, 2021; Wolff et al., 2014). By extension, and considering the shared mitochondrial and nuclear assignments of hybrids are consistently associated with (opposite) external morphology, the process of hybridization may play a significant role in deer speciation within North America. This is also loosely supported by the fact that Coues WTD mitochondrial haplotypes are more closely related to MD haplotypes although they retain clear morphological differences by species (Figure 2). Under this scenario, non‐compatible mito‐nuclear combinations would be rapidly purged from populations, and as such, hybridization may not lead to substantial fitness consequences for wild populations. However, in small (and declining) populations, such as with MD, excessive hybridization may result in further decline if a moderate or high percentage of hybrids from costly breeding efforts are non‐viable.

In theory, the rapid westward expansion of WTD over the past century may be facilitated by hybridization through various mechanisms (Pfennig et al., 2016). This could result in asymmetrical introgression of neutral genes from non‐hybrid MD into the invading species (Barton & Hewitt, 1989) as has been reported in a wide range of other species (Currat et al., 2008; Gantenbein & Largiadèr, 2002; Leaché, 2011; Pierce et al., 2017). Or, it may lead to introgression of adaptive genotypes (e.g., mito‐nuclear combinations) from MD into WTD (and vice versa) that would allow for greater fitness within peripheral environments (Pfennig et al., 2016).

### 4.4 Management implications and future directions

Our study, along with other recent related work (Chafin et al., 2021; Russell et al., 2019), constitutes a relatively new chapter in deer genetics research that can now address an old observation of interspecific hybridization with more powerful analytical methods. Yet, the results we have presented pose as many new questions and problems as they address and undoubtedly will precipitate productive future research avenues with broader geographic scope. Foremost, given the vast economic and cultural importance of North American deer and imminent threats from emerging disease and continuing population trends, it is critical that land managers, scientists, and society contribute to building comprehensive geographic voucher representation within public museum archives (Cook & Light, 2019). Existing DNA extractions and limited tissues are ephemeral and may be rapidly lost when housed in research laboratories and government offices. Publicly archived specimens accompanied by skeletal voucher materials can more effectively be maintained in perpetuity and combined from multiple collectors and timeframes for more comprehensive perspectives. Given that deer skulls are often valued by hunters, if skull materials are not available, we recommend collection of the hind hock and hoof, which would provide skeletal and skin elements that are diagnostic among species and potentially useful for quantification of intermediate hybrid morphology, including astragalus bones and metatarsal glands.

One research avenue that greater sample coverage would enable is a range‐wide comparative population genomic analysis of all *Odocoileus* deer lineages that should verify or refute the convoluted evolutionary history of these species as indicated by their matrilineal history and accurately diagnose the distribution of genetic diversity across North America. Related to this would be greater sampling across what are evidently complex mosaic zones of hybridization to establish with more confidence whether gene flow among MD, WTD, and BTD is a predictable process, whether introgression of genetic material across species is progressive, or functionally limited, or some combination depending on, for instance, functional or neutral genomic regions. This would also aid in understanding the geographic limits for hybridization as related to the recognized zone of sympatry between MD and WTD. Finally, movement of deer through dispersal or range expansion can be strongly influenced by land forms, which in turn can dictate the spread of both genes (through hybridization) and disease. Assessing relative connectivity of deer populations across broad landscapes through use of genomic data would identify land‐use practices that differentially influence deer movement and contribute to more effective adaptive management at the population level.

Genomic sequencing via ddRADseq methods provides much improved insight toward evolutionary processes compared with only one or a few loci, in relation to assessment of genetic diversity, demography, phylogenetic reconstruction, and estimation of gene flow. However, these data are still limited in their ability to address questions of gene function in response to changing environments or adaptive evolution. Targeted gene sequencing approaches, including exome capture, transcriptome sequencing, or whole‐genome sequencing, would provide lucrative opportunities for investigating the effects of hybridization between MD and WTD from a functional perspective in three key areas, including (1) a more thorough scan for genes under different forms of selection to relate critical life functions to environmental variables, and fitness consequences for deer in different regions; (2) identification of genomic regions or gene combinations that are consistently maintained or purged through the generation of hybrids and how the process of multi‐generational backcrossing can influence the integrity of species boundaries; and (3) assess the genomic underpinnings of susceptibility to diseases, in particular with respect to multi‐genic CWD susceptibility, and how the distribution of disease resistance is related to landscape features and biogeography, but also to hybrid zones and hybrid fitness.

Hybridization of WTD and MD in Kansas is evidently not rare, at least within the regions studied. These regions were also explicitly the focus of our research due to concern for continued observed population trends, as well as increasing incidence of CWD. We have demonstrated that population trends (decline in MD and expansion in WTD) are reflected in estimates of population genetic diversity. These trends may also substantially promote the incidence of hybridization. Importantly, we have shown that genomic introgression in both directions between species exhibits potentially predictable associations of mitochondrial and nuclear assignment as well as consistent levels of genomic mixing. This suggests a mechanism for maintaining at least some viable hybrids that likely promotes distinction of species boundaries. Considering increasing focus on genomic interactions among these species, we expect that ongoing investigations will provide valuable insight toward management of these key game species across a continent‐wide zone of sympatry.

## CONFLICT OF INTEREST

The authors declare no conflict of interest.

## Supporting information

Fig S1‐S2

Table S1

## Data Availability

Raw sequence data are available via the NCBI Sequence Read Archive (SRA) under BioProject PRJNA783357 and cytochrome b sequences are under the accession numbers OK668083‐668151. Assembled data and scripts are openly available in Figshare (https://doi.org/10.6084/m9.figshare.17097062.v1).
