# Supplementary material for: Population genomics of free‐ranging Great Plains white‐tailed and mule deer reflects a long history of interspecific hybridization
Source: Evol Appl. 2021 Dec 14;15(1):111–31. doi: 10.1111/eva.13330 (PMC8792484; doi:10.1111/eva.13330)

Supplementary material

**Title: Population genomics of free-ranging Great Plains white-tailed and mule deer reflects a long history of inter-specific hybridization**

**Fig. S1**. Outlier loci identified (red) for white-tailed deer and mule deer by BayeScan. X-axis: log10(q values) for all SNPs, the threshold is −1. Y-axis: FST-values, where high values indicate directional selection, low values balancing selection.


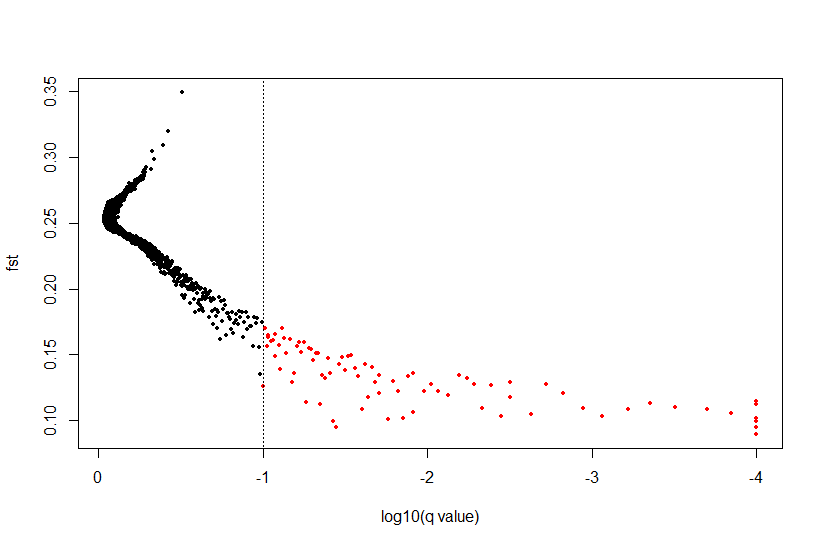


**Fig S2**. Scree plot of eigen values of principal components for K 1-10, elbow of the curve at K=2 indicates the number of clusters retained.


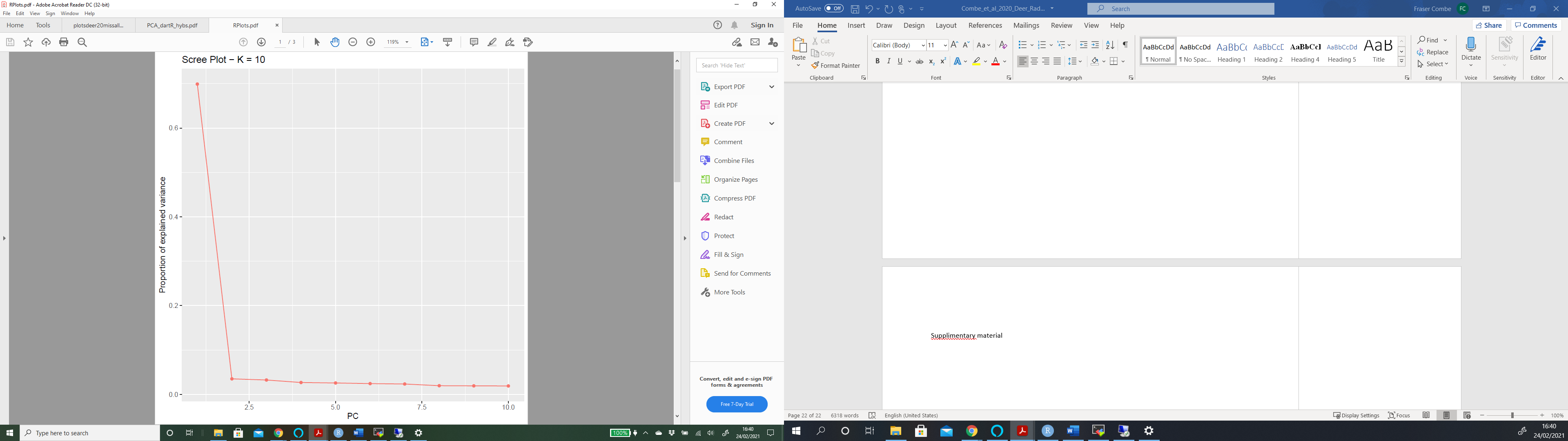

Supplement: Supplementary file 1 — Fig S1‐S2 [file EVA-15-111-s001.docx]
